# Urinary tract infection‐related delirium in Alzheimer's disease and related dementias: Clinical challenges and translational opportunities

**DOI:** 10.1002/alz.71184

**Published:** 2026-02-03

**Authors:** Sarah Kim, Sarah Kremen, Itai Danovitch, Shouri Lahiri

**Affiliations:** ^1^ Department of Neurology Cedars‐Sinai Medical Center Los Angeles California USA; ^2^ Department of Psychiatry and Behavioral Neurosciences Cedars‐Sinai Medical Center Los Angeles California USA; ^3^ Department of Neurosurgery Cedars‐Sinai Medical Center Los Angeles California USA; ^4^ Department of Biomedical Sciences Cedars‐Sinai Medical Center Los Angeles California USA

**Keywords:** Alzheimer's disease, Alzheimer's disease and related dementias, delirium, dementia, psychiatry, urinary tract infection, UTI

## Abstract

Alzheimer's disease and related dementias (ADRD) affects millions of patients worldwide and is a leading cause of morbidity in older adults. Patients with ADRD are particularly susceptible to developing urinary tract infection (UTI) and UTI‐related delirium, creating a self‐perpetuating cycle in which ADRD increases vulnerability to infection and delirium, while delirium itself accelerates cognitive and functional decline. This review summarizes the epidemiology, clinical implications, and diagnostic guidelines for UTI and UTI‐related delirium in ADRD. It also examines emerging biological mechanisms, including interleukin‐6–mediated pathways, and discusses evidence‐based strategies for prevention and management.

## Highlights

- Individuals with Alzheimer's disease and related dementias (ADRD) are highly susceptible to developing urinary tract infection (UTI) and UTI‐related delirium.
- Delirium increases risk of incident ADRD and accelerates cognitive decline in patients with ADRD.
- UTI is challenging to diagnose in patients with ADRD and/or delirium due to atypical or absent genitourinary symptoms.
- Prompt initiation of antimicrobial therapy for UTI may improve delirium, ADRD, and survival outcomes.
- Emerging therapies for UTI‐related delirium target interleukin‐6 (IL‐6) trans‐signaling and hormonal neuroprotection.

## 1 BACKGROUND

Alzheimer's disease (AD) and related dementias (ADRD) affects millions of patients worldwide and is a leading cause of morbidity in older adults. Patients with ADRD are particularly susceptible to developing urinary tract infection (UTI) and UTI‐related delirium, creating a self‐perpetuating cycle in which ADRD increases vulnerability to UTI and delirium, while delirium itself accelerates cognitive and functional decline. Diagnosis of UTI in this population is challenging, as patients often cannot report focal genitourinary symptoms such as dysuria or urinary urgency. As a result, delays in diagnosis and treatment are common, contributing to poor health outcomes.

Despite their frequent coexistence, the impact of UTI on the course of ADRD, including long‐term outcomes and risk of progression, remains poorly defined. This review examines the epidemiology, clinical implications, and current guidelines for diagnosis of UTI and delirium in ADRD. We further report putative biological mechanisms that underlie the pathogenesis of UTI to delirium in ADRD and provide evidence‐based strategies for clinical management.

## 2 METHODS

We conducted a structured narrative review to synthesize current evidence on the relationships among UTI, delirium, and ADRD. A comprehensive literature search was performed in PubMed using the following terms: (“dementia” OR “Alzheimer's disease”) AND “urinary tract infection”. The search included English‐language articles published through October 2025. We considered both clinical and preclinical studies. A total of 433 abstracts were identified in the search. Titles and abstracts were screened to identify studies addressing at least two of the three key concepts (UTI, delirium, ADRD). Full texts of eligible articles were then reviewed to evaluate reported evidence for causal, contributory, or associative relationships. Studies that did not address cognitive or neurobehavioral outcomes were excluded. Findings were synthesized qualitatively, with attention to study design, population, and mechanistic insights relevant to interactions among infection, delirium, and neurodegeneration.

## 3 EPIDEMIOLOGY, RISK FACTORS, AND CLINICAL IMPLICATIONS

### 3.1 ADRD and UTI

ADRD affects over 57 million people globally and imposes unsustainable healthcare costs exceeding US$1.3 trillion annually.^1^ Up to 50% of patients with ADRD visit the emergency department every year and are most frequently discharged with a diagnosis of UTI.^2^, ^3^ ADRD and UTI share several common clinical risk factors including advanced age and female sex, with women accounting for two‐thirds of all patients with Alzheimer's disease and UTIs occurring 30 times more frequently in women than men.^3^, ^4^, ^5^, ^6^, ^7^, ^8^, ^9^, ^10^, ^11^ Additional age‐related changes common in ADRD including diabetes, poor bladder control leading to urinary retention or incontinence, constipation, malnutrition, vaginal atrophy in the postmenopausal state, prostate hyperplasia, and immobility further increase susceptibility to UTI.^5^, ^6^, ^7^, ^8^, ^12^, ^13^, ^14^ A recently published scoping review by Wu et al.^15^ identified 14 UTI risk factors in people living with dementia, organized across individual (biological, behavioral), caregiver‐patient relationship, community, and societal levels. In addition to physiological vulnerabilities related to aging and cognitive impairment, the framework highlights the central role of caregivers and healthcare system interactions as modifiable determinants of UTI risk in dementia, with potential relevance for the prevention of UTI‐related delirium.

The consequences are significant. UTI is the most common cause of infection in elderly women in both hospital and long‐term care settings, and the second most common infection in older women living in the community.^16^, ^17^, ^18^ Patients with ADRD who present to the ED are more likely to be admitted to the hospital, with frequent readmissions, longer hospitalization and hospital‐acquired complications, as well as increased mortality.^19^, ^20^, ^21^, ^22^ UTIs are responsible for 25% of all geriatric hospitalizations,^14^ while untreated or undertreated UTI can lead to systemic infection and sepsis with multi‐organ damage.^23^ In a retrospective cohort study, the incidence of mortality 60 days following UTI diagnosis in patients with ADRD was nine per 1000 person‐years (incidence rate ratio [IRR] = 6.31, 95% confidence interval [CI] [3.82‐10.44], *p*‐value < 0.001), with lower 60‐day survival rate (hazard ratio [HR] = 1.34 [1.31‐1.38], *p*‐value < 0.005).^4^ The study also highlighted that treatment timing significantly influenced survival rate, with deferred and withheld treatment being associated with higher risk of 60‐day mortality compared to immediate treatment (deferred: HR = 1.57, 95% CI [1.22‐2.02], *p* value < 0.001; withheld: HR = 1.85, 95% [CI 1.63‐2.09], *p* value < 0.001).

Despite the high stakes, diagnosis of UTI in patients with ADRD remains challenging. Standard diagnostic criteria rely on self‐reported genitourinary symptoms, such as dysuria or urgency, which many patients with ADRD cannot communicate either due to cognitive impairment or altered sensory perceptions. These barriers, often compounded by superimposed delirium, contribute to delays in diagnosis and treatment, leading to worse clinical outcomes (Table 1).

**TABLE 1:** Key clinical implications of UTI in patients with ADRD.

Diagnostic challenges	Cognitive impairment and/or language/communication impairment to report focal genitourinary symptoms
	Alterations in sensory perception
	Overlapping symptoms with ADRD (e.g., incontinence)
	Atypical features (e.g., fatigue, anorexia, dizziness, and falls)
Healthcare burden	Frequent ED visits
	High rate of admission and readmission
	Longer hospitalization
Medical complications	Sepsis
	Multi‐organ damage
	Delirium/acceleration of underlying neurodegenerative disorder
	Increased mortality

Abbreviations: ADRD, Alzheimer's disease and related dementias; ED, emergency department; UTI, urinary tract infection.

### 3.2 UTI‐related delirium and ADRD

Delirium and ADRD are closely interconnected, sharing overlapping risk factors, pathophysiology, and clinical manifestations. While delirium is an acute, sometimes reversible syndrome characterized by inattention, short‐term memory impairment, or executive dysfunction, ADRD represents a chronic, progressive, and irreversible decline across multiple cognitive domains.^24^, ^25^, ^26^ When mild or short‐lasting, delirium may be fully reversible; however, severe or persistent delirium can lead to long‐term ADRD phenotypes.^27^, ^28^, ^29^ Indeed, delirium and ADRD may represent points along a continuum of brain vulnerability, somewhat analogous to how acute coronary syndromes can accelerate the trajectory toward chronic heart failure.

Epidemiological studies consistently support this bidirectional relationship. ADRD is a leading risk factor for delirium,^30^, ^31^ but delirium itself independently increases the risk of cognitive decline and incident ADRD.^24^, ^27^, ^29^, ^32^, ^33^, ^34^, ^35^, ^36^, ^37^ A meta‐analysis of 23 studies found delirium to be an independent risk factor for long‐term cognitive decline.^27^ One prospective cohort study reported an increased risk of new dementia diagnosis at one‐year follow‐up in patients hospitalized with delirium (OR = 8.8, 95% CI [1.9‐41.4]), with recurrent or prolonged (> 5 days) delirium conferring worse cognitive outcomes.^35^ Another study demonstrated that cognitive decline in the delirium group proceeded at 2.2 times the rate in patients without delirium in the following year.^36^ One large retrospective cohort study of over 650,000 patients aged ≥65 years demonstrated not only the rate of incident dementia in delirium group to be 3.4 times higher than the no‐delirium group, but a dose‐dependent relationship between the number of episodes of delirium and incident ADRD risk, with each additional episode of delirium contributing to a 20% increased risk of dementia (HR = 1.20, 95% CI [1.18‐1.23]).^32^ Another population‐based cohort study demonstrated worse Clinical Dementia Rating score (OR = 3.1, 95% CI [1.5‐6.3]), and worse global function score (OR = 2.8, 95% CI [1.4‐5.5]) at follow‐up in up to 10 years, as well as a loss of one point on Mini‐Mental State Examination per year (95% CI [0.11‐1.89]) in the delirium group compared to the non‐delirium group.^37^

Collectively, these findings highlight delirium not only as a marker of vulnerability but also as a driver of dementia progression. Preventing delirium and minimizing recurrent episodes may therefore represent one of the most effective, yet underutilized, opportunities to reduce ADRD incidence and slow cognitive decline. Although few studies have directly evaluated UTI‐related delirium as a driver of ADRD progression or outcomes, converging epidemiological, clinical, and mechanistic evidence supports a coherent framework linking these conditions. UTI is not only highly prevalent in patients with ADRD but also frequently precipitates delirium, which in turn is independently associated with accelerated cognitive decline and increased incident dementia risk. Future prospective studies are needed to examine the impact of UTI and UTI‐related delirium on ADRD outcomes and to evaluate underlying modifiable biological mechanisms.

## 4 PATHOPHYSIOLOGY

### 4.1 UTI‐related delirium and ADRD

Recent preclinical studies demonstrate that interleukin‐6 (IL‐6) trans‐signaling may underlie the pathogenesis of UTI‐related delirium.^12^, ^38^, ^39^ In response to UTI, IL‐6 is released into the systemic circulation and binds to the soluble IL‐6 receptor to form a complex that crosses into the brain, where it activates the glycoprotein 130 receptor on neurons. This cascade induces increased cleaved caspase‐3 expression, a marker of neuronal injury and neurodegeneration.^40^, ^41^, ^42^ These findings provide a biologically plausible mechanism by which UTI may precipitate acute delirium and ADRD. While these studies do not provide a specific link between UTI and ADRD, they rather implicate a process by which acute systemic inflammation from UTI may lead to neuronal injury, neuroinflammation, and neurodegeneration. Supporting this, chronic elevations in plasma IL‐6 have been associated with increased risk of AD or Parkinson's disease,^12^, ^43^, ^44^, ^45^, ^46^ underscoring the role of peripheral‐central immune interactions in neurodegenerative conditions.

## 5 DIAGNOSTIC CHALLENGES

### 5.1 UTI, delirium, and ADRD: Epidemiologic and diagnostic complexity

Perhaps surprisingly, the role of UTI as a trigger for delirium remains controversial. Indeed, observational studies report high rates of co‐occurrence: in a study of patients with delirium, UTI rates ranged from 25.9% to 32% compared with 13% in those without delirium; and in patients with UTI, delirium rates ranged from 30% to 35% compared with 7.7% to 8% in those without UTI.^3^ Another study implicated UTI in nearly 40% of hospitalized patients with delirium.^36^ In contrast, Infectious Disease practice guidelines require focal genitourinary symptoms to diagnose UTI—without exceptions for patients with ADRD or delirium who may be unable to communicate these symptoms. These recommendations are based largely on trials showing no benefit of antimicrobial therapy in patients with “asymptomatic bacteriuria,”^47^, ^48^, ^49^ along with concerns about antimicrobial resistance.^50^, ^51^, ^52^, ^53^, ^54^, ^55^ Consequently, many patients with delirium or ADRD who do not report focal genitourinary symptoms are diagnosed with asymptomatic bacteriuria rather than UTI, leading to delayed or missed treatment opportunities. A key point is that current practice guidelines base their recommendations on studies of antimicrobial therapy in patients with asymptomatic bacteriuria rather than in patients with delirium as the isolated presumed sign or symptom of UTI.^56^, ^57^

### 5.2 Identifying UTI‐related delirium in ADRD

#### 5.2.1 UTI in ADRD

Current Infectious Disease guidelines recommend that in cognitively impaired, bacteriuric patients without focal urinary symptoms, clinicians should search for alternative causes and observe rather than initiate antimicrobial therapy.^58^, ^59^ This recommendation is justified by a lack of a known causal relationship between bacteriuria and delirium and benefit with antimicrobial therapy in patients without focal genitourinary symptoms.^60^

In practice, however, patients with ADRD may not report genitourinary symptoms either due to cognitive impairment, language or hearing difficulties, altered sensory perceptions, or superimposed UTI‐related delirium.^14^, ^61^, ^62^ Reliance on patient‐reported symptoms therefore creates a major barrier to accurate and timely diagnosis. Moreover, UTI presentation in this population is frequently atypical: in one prospective cross‐sectional cohort study, fever was present in only 11% of the elderly who participated in the study whereas delirium was the most common atypical feature (28.9%).^13^ These diagnostic challenges contribute to frequent delays or missed diagnoses (Table 1).

However, these considerations must be balanced with the reasonable concern for overdiagnosis of UTI and inappropriate or excessive use of antibiotics, which can contribute to *Clostridium difficile* infection and increased antibiotic resistance patterns. Moreover, there is also evidence that the use of certain antibiotics, such as cefepime, may contribute to an increased risk of delirium.^63^, ^64^, ^65^ Recent preclinical data suggesting that UTI‐induced delirium may be attenuated by antimicrobial therapy provide a strong rationale for carefully designed randomized clinical trials in patients with delirium as the isolated presumed symptom or sign of UTI.^66^

#### 5.2.2 Delirium in ADRD

When delirium is suspected as a manifestation of UTI in patients with ADRD, it is critical to distinguish acute delirium from chronic ADRD‐related cognitive dysfunction. The *Diagnostic and Statistical Manual of Mental Disorders, 5th edition* (DSM‐5) from the American Psychiatric Association^67^ and the *International Statistical Classification of Diseases and Related Health Problems, 10th revision* from the World Health Organization^68^ provide the reference standards for delirium diagnosis, while clinical tools such as the Confusion Assessment Method (CAM) and the 4AT are widely used for both screening and trending the course of delirium.^69^ The key distinguishing factor between delirium and ADRD is that delirium is acute, fluctuating and sometimes reversible, whereas ADRD is insidious, progressive and without cure to date, as summarized in Table 2.^28^, ^29^

**TABLE 2:** Key distinguishing features of delirium and dementia.

Feature	Delirium	Dementia
Onset	Sudden, abrupt	Insidious, gradual (TBI^*^ and vascular dementia^*^)
Etiology	Underlying medical condition, substance intoxication/withdrawal, medication side effect	Underlying neurodegenerative process
Duration	Hours to days (though can be prolonged in some cases)	Months to years
Course	Reversible; fluctuating, remitting	Progressive (TBI^*^ and vascular dementia^*^)
Attention	Reduced ability to focus, sustain or shift attention is a hallmark feature occurring early in presentation	Usually normal until progresses to severe dementia (Lewy Body Dementia^**^)
Alertness/consciousness	Fluctuating	Generally intact (Lewy Body Dementia^**^)
Speech	Incoherent, disorganized, distractible	Ordered, though may develop anomia or aphasia

*Note*: There may be overlapping symptoms between the two, and they may co‐exist in an individual patient. Comparative features of delirium and dementia.

Abbreviation: TBI, traumatic brain injury.

* May have abrupt onset and static course.

** One of the hallmark features of Lewy body dementia is fluctuation in attention/alertness.

Despite being common in patients with ADRD, delirium remains under‐recognized: even as of 2015, approximately 60% of cases went undetected by treating clinicians and nurses.^70^, ^71^, ^72^ This is, in part, because delirium is phenotypically heterogenous, often subtle in presentation, and may overlap with features of dementia. Delirium is also primarily a clinical diagnosis, relying on history taking and observation by healthcare professionals, rather than radiological or serologically‐assisted diagnoses.^73^ The key feature in distinguishing delirium from dementia remains an acute change in mentation from baseline, which is often best observed by caregivers and healthcare professionals in the community. Seeking medical attention to rule out acute medical illnesses such as UTI is recommended for any acute change or worsening of mood disturbance, behavioral issues or psychosis in patients with ADRD in the community.

Future research is needed to refine diagnostic tools that facilitate differentiation of acute delirium from chronic ADRD, both for clinicians and caregivers in community settings. Recent technological advances in smartphone‐based platforms and applications that monitor objective changes in both cognitive and physical function in patients with ADRD may be helpful in early detection and treatment of UTI‐related delirium.^74^ Finally, diagnostic paradigms that incorporate measurements of systemic inflammatory markers, for example, increased plasma IL‐6, and neural injury markers, for example, S100β, alongside clinical observation may ultimately increase sensitivity and specificity for diagnosing UTI‐related delirium.

## 6 MANAGEMENT

### 6.1 Antimicrobial therapy

Antimicrobial therapy remains the mainstay management strategy for UTI‐related delirium; however, it is worth emphasizing that antibiotics only serve to prevent UTI‐related brain dysfunction and do not address what brain dysfunction has already occurred, potentially due to delayed diagnosis and treatment.^66^ This is consistent with clinical experience where delirium can take days or even weeks to resolve after a UTI has cleared, particularly when there has been a delay to diagnosis and initiation of antimicrobial therapy. Thus, early diagnosis and treatment of UTI provides the best opportunity to improve delirium outcomes. Additionally, it is critical that time to antimicrobial therapy initiation and duration of delirium be considered as key factors in any clinical trial that endeavors to evaluate potential beneficial effects of antimicrobial therapy.

**FIGURE 1:**
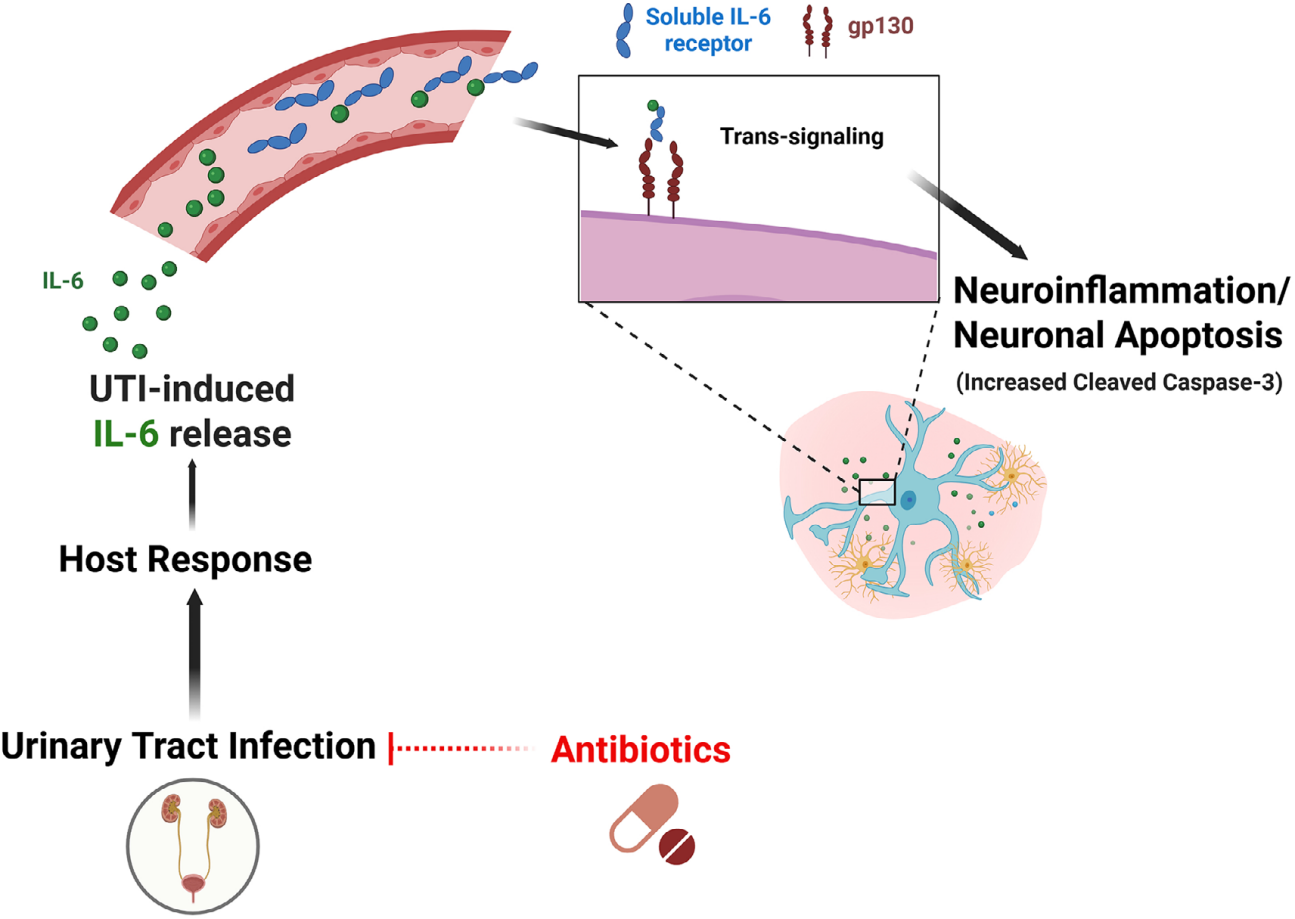
Urinary tract infection induces host IL‐6 response. IL‐6 complexes with the soluble IL‐6 receptor in the periphery and initiates IL‐6 trans‐signaling via the glycoprotein 130 transmembrane (gp130) receptor on neurons, resulting in neuroinflammation and neuronal apoptosis. Antimicrobial therapy attenuates IL‐6 trans‐signaling via mitigation of urinary tract infection, preventing subsequent brain injury. IL‐6 IK‐6, interleukin‐6.

Figure 1 illustrates a biologically plausible pathway underlying UTI‐related delirium—UTI induces the host response, that is, increased IL‐6/soluble IL‐6 receptor, followed by trans‐signaling on neurons, culminating in delirium. Future studies are required to evaluate therapeutics that address biological mechanisms downstream of the infection source, such as the host IL‐6 response or direct neuroprotectants.

### 6.2 Hormonal therapy

Topical vaginal estrogen has been demonstrated to significantly reduce the incidence of recurrent UTI in postmenopausal women by inducing proliferation of urothelial cells, reducing vaginal mucosa atrophy, lowering vaginal pH, and reducing rates of incontinence.^75^, ^76^ In a recent preclinical study, systemic 17β‐estradiol improved UTI‐induced delirium phenotypes in postmenopausal mice via attenuation of IL‐6 trans‐signaling and direct neuroprotection.^46^ While clinical trials are required to confirm this finding in patients, it may be reasonable, after careful consideration of potential risks and benefits, to consider support for systemic hormone replacement for postmenopausal women with recurrent UTI‐related delirium who may have an additional strong indication for systemic hormone replacement, such as hot flashes. Though past recommendations did not support a role for chronic hormone replacement out of concern for increased rate of stroke, pulmonary embolism and other adverse health events, there has been a recent resurgence of interest in hormone replacement therapy for postmenopausal women, prompted by analysis of prior data suggesting fewer adverse effects than previously thought.^77^, ^78^, ^79^, ^80^, [Table alz71184-tbl-0003]

### 6.3 Low‐risk adjunctive treatments with modest clinical evidence

Probiotics, in particular *lactobacillus*, inhibit the growth of uropathogens and enhance the immune response, potentially reducing recurrent UTIs.^70^, ^81^, ^82^ Other supplements such as cranberry products, ascorbic acid (vitamin C), and D‐mannose are also widely used to prevent recurrent UTI, though support for their use is not as well established in clinical studies. Regardless, a few studies have reported beneficial effects of cranberry products,^71^, ^72^ and D‐mannose,^73^, ^83^, ^84^ and the potential health‐related harm from these interventions are likely to be low relative to potential benefits. Emerging strategies for UTI prevention include vaccines against uropathogenic organisms^85^, ^86^, ^87^, ^88^ and methenamine.^89^ Prophylactic use of methenamine, an antibiotic‐sparing antiseptic agent, has been shown to reduce risk of recurrent UTI.^90^, ^91^ Recommendations for prevention of UTI are summarized in Table 3.^92^, ^93^, ^94^ In addition, a recent dementia‐specific management framework proposed promoting physical activity, ensuring adequate hydration, optimizing bathroom design, and reducing caregiver stress to further support UTI prevention.^15^

**TABLE 3 alz71184-tbl-0003:** Key management strategies of UTI‐related delirium

Parameter	Intervention	Class of recommendation; level of evidence
Prevention of UTI/recurrent UTI^*^	Topical hormonal therapy	Moderate recommendation; evidence level: Grade B
	Cranberry products	Moderate recommendation; evidence level: Grade B
	D‐mannose	Moderate recommendation; evidence level: Grade B
	Methenamine	Conditional recommendation; evidence level: Grade C
	Antibiotic prophylaxis	Conditional recommendation; evidence level: Grade B
	Timely, targeted antimicrobial therapy	Strong recommendation; Grade A (for UTI)
	Probiotics	Clinical review
Nonpharmacologic intervention^**^	Multicomponent approach targeting sleep, sensory impairment and dehydration	Strong recommendation; evidence level: Grade A/B
	Early mobilization	Moderate recommendation; evidence level: Grade B
Pharmacologic intervention^**^	Antipsychotics	Moderate/weak recommendation; evidence level: Grade B/C
	Antidepressants	Clinical review
	Cholinesterase inhibitors	Clinical review

*Note*: Management of UTI‐related delirium. Nonpharmacologic interventions are recommended before pharmacologic interventions and can also help prevent delirium. Pharmacologic treatments, especially antipsychotics, are reserved for agitation in delirium.

Abbreviation: URI, urinary tract infection.

* Adapted from American Urological Association guidelines for prevention of UTI/recurrent UTI in women (https://www.auanet.org/guidelines‐and‐quality/guidelines/recurrent‐uti ) and Infectious Diseases Society of America and the European Society for Microbiology and Infectious Diseases guidelines for uncomplicated cystitis and pyelonephritis (Clinical Infectious Diseases, 2011).

** Adapted from American Geriatrics Society (*J Am Geriatr Soc* 2015), and American Psychiatric Association guidelines for prevention and treatment of delirium (https://www.psychiatry.org/news‐room/news‐releases/apa‐published‐updated‐guideline‐for‐delirium).

N.B. As few interventions have been evaluated in UTI‐related delirium, these recommended management strategies draw on evidence from UTI and delirium studies. These recommendations are intended to guide care at the UTI‐delirium interface, for example, prevention of UTI/recurrent UTI for patients with recurrent UTI‐related delirium episodes or delirium‐directed interventions for clinical situations where delirium has already occurred.

### 6.4 Management of UTI‐related delirium

Delirium management centers on treating the underlying cause and implementing preventative strategies.^25^, ^95^ Primary prevention of delirium with nonpharmacologic interventions is gaining widespread acceptance as an effective strategy to mitigate delirium in older adults.^26^, ^96^, ^97^ These nonpharmacologic strategies include reorientation, addressing sensory impairments such as vision and hearing loss, environmental modifications to reduce overstimulation and institute circadian rhythm, and behavioral interventions to increase mobility and decrease use of physical restraints.^31^, ^94^, ^98^, ^99^, ^100^, ^101^, ^102^ The most widely implemented approach is the Hospital Elder Life Program (HELP), a multicomponent intervention strategy targeting risk factors for delirium, which includes reorientation, therapeutic activities, reduction of psychoactive medications, early mobilization, promoting circadian health, hydration and nutrition maintenance, and provision of vision and hearing adaptations.^103^, ^104^, ^105^

In a large‐scale controlled clinical trial evaluating HELP, delirium developed in 9.9% of the intervention group, compared with 15.0% of the usual‐care group (matched OR 0.60, 95% CI [0.39‐0.92]).^31^ HELP was shown to be cost‐effective and intervention‐effective in diverse settings and populations in a large scale controlled clinical trial as well as in over 10 follow‐up studies.^106^, ^107^, ^108^ Such non‐pharmacologic strategies can not only prevent delirium but also help with the treatment of delirium. However, barriers to implementing and sustaining such interventions include constraints on resources or availability of skilled interdisciplinary professionals.

The role of pharmacologic strategies in delirium remains controversial and an active area of investigation. In general, though the use of antipsychotics has not been supported for prevention or treatment of delirium, they are often used in clinical practice for the management of delirium agitation.^109^, ^110^, ^111^ Haloperidol has been shown to reduce the incidence of delirium in a small group of post‐operative patients,^112^ but the result was not confirmed in a larger study.^113^ Moreover, neuroleptic agents, especially first‐generation antipsychotics such as haloperidol, carry a higher rate of extrapyramidal side effects and acute dystonia. In contrast, atypical antipsychotics, such as olanzapine, quetiapine, risperidone, carry a lower rate of extrapyramidal side effects. However, both atypical antipsychotics and parenteral haloperidol are associated with an increased risk of stroke in elderly patients with dementia, and contribute to prolongation of the QT interval.^114^ Furthermore, there is no data to demonstrate any verifiable advantage of one antipsychotic agent over another.^115^ Accordingly, careful consideration of potential risks and benefits of these medications is justified prior to clinical use.

Other potential pharmacologic treatments for delirium include cholinesterase inhibitors and 5‐HT receptor antagonists, such as trazodone. Several case reports and one open‐label study have suggested promising results from cholinesterase inhibitors,^116^, ^117^, ^118^, ^119^ but other randomized controlled trials have shown no benefit.^120^, ^121^ The utility of cholinesterase inhibitors for treatment of delirium agitation and improvement in duration of delirium warrants further examination in the context of the bidirectional relationship between delirium and dementia, as they also play a role in improving behavioral and psychological symptoms of dementia and mitigating cognitive decline in ADRD.^122^, ^123^, ^124^, ^125^ Benzodiazepines are generally not recommended as a first‐line treatment as they can exacerbate mental status changes and cause oversedation. Overall, minimizing the use of deliriogenic agents such as benzodiazepines, opioids, and anticholinergics is recommended.^29^, ^69^, ^126^, ^127^, ^128^, ^129^, ^130^ Alternatives to benzodiazepines such as gabapentin and dexmedetomidine are being actively investigated.^131^, ^132^ The side effect profile of any medication regimen should be carefully reviewed for each patient, with ongoing risk‐benefit analyses. Any pharmacologic agent chosen should be initiated at the lowest starting dose, cautiously titrated, adjusted with monitoring of response and any adverse events, and promptly tapered as delirium improves. Nonpharmacologic and pharmacologic strategies for management of UTI‐related delirium are summarized in Table 3.

## 7 CRITICAL APPRAISAL OF EVIDENCE BASE

There are important limitations in the evidence base for UTI‐related delirium in ADRD that warrant consideration. The predominantly observational literature often variably defines UTI and asymptomatic bacteriuria, particularly in the context of delirium, while delirium itself is inconsistently ascertained using validated instruments. Furthermore, most studies do not specifically address UTI‐related delirium in ADRD and rather tend to focus on these conditions individually. Accordingly, although converging epidemiological, clinical, and mechanistic data link UTI‐related delirium to adverse cognitive trajectories in ADRD, causal inferences and directionality remain provisional until results from rigorously designed prospective studies incorporating standardized definitions of UTI, systematic delirium/ADRD phenotyping, and biological biomarkers become available.

## 8 CONCLUSION

UTI is among the most frequent systemic illnesses in older patients and occurs disproportionately in people with or at risk for ADRD. Up to one‐third of patients with UTI develop delirium, which not only complicates acute care, but also heralds incident or accelerated progression of ADRD. Delirium may therefore represent both a prodromal manifestation along the ADRD spectrum and a marker of decompensation in a brain with underlying vulnerability.

Emerging preclinical studies implicate IL‐6 trans‐signaling as a potential mechanistic driver of UTI‐related delirium, highlighting peripheral–central immune crosstalk as a therapeutic target. Clinically, a strategy emphasizing early recognition and prompt initiation of antimicrobial therapy remains prudent to mitigate the deleterious impact of UTI on delirium, ADRD progression, and mortality, pending confirmation from prospective clinical trials.^133^ Future research must focus on developing sensitive diagnostic tools for UTI in cognitively impaired patients who cannot reliably report genitourinary symptoms, and on designing targeted therapies that not only eradicate infection but also prevent or attenuate UTI‐related delirium.

## AUTHOR CONTRIBUTIONS

Conceptualization: Shouri Lahiri, MD, Writing: Sarah Kim, MD and Shouri Lahiri, MD, Critical revisions: Sarah Kim, MD, Sarah Kremen, MD, Itai Danovitch, MD, Shouri Lahiri, MD. All authors revised the manuscript critically for important intellectual content. All authors read and agreed with the final manuscript.

## FUNDING

F. Widjaja Foundation (SL).

## CONFLICT OF INTEREST STATEMENT

The authors declare no conflicts of interest relevant to this work. Author disclosures are available in the Supporting Information.

## Supporting information



Supporting Information
